# Editorial: Current challenges in complement diagnostics

**DOI:** 10.3389/fimmu.2023.1334050

**Published:** 2023-11-24

**Authors:** Per H. Nilsson, Lillemor Skattum, Erik J. M. Toonen

**Affiliations:** ^1^ Linnaeus Centre for Biomaterials Chemistry, Linnaeus University, Kalmar, Sweden; ^2^ Department of Chemistry and Biomedical Sciences, Linnaeus University, Kalmar, Sweden; ^3^ Clinical Immunology and Transfusion Medicine, Region Skåne, Lund, Sweden; ^4^ Department of Laboratory Medicine, Section of Microbiology, Immunology and Glycobiology, Lund University, Lund, Sweden; ^5^ Research & Development Department, Hycult Biotech, Uden, Netherlands

**Keywords:** complement diagnostics, complement system, assessment of complement components, diagnostic techniques and procedures, complement-mediated disease

The complement system is an essential innate immune surveillance network that plays a crucial role in safeguarding the host against various threats, including invading microorganisms, dying or malignant cells, and immune complexes. It is a highly intricate system, comprising about 50 soluble and cell surface-bound proteins that interact with each other to eliminate danger, regulate cell activity, and retain homeostasis. To prevent uncontrolled activation, the complement system needs tight regulation. Dysregulation or inadequate functioning is associated with a myriad of diseases, including autoimmune and acute or chronic inflammatory diseases, infection susceptibility, and cancer. Uncontrolled or exaggerated activation can lead to life-threatening conditions such as systemic inflammation, shock, and, in the worst cases, organ failure and death (1).

In recent years, the complement system has been the subject of significant research interest as a target for therapeutic intervention. Eculizumab, the first approved complement inhibitor, has been highly effective in treating diseases associated with complement dysregulation such as atypical hemolytic uremic syndrome (aHUS) and paroxysmal nocturnal hemoglobinuria (PNH), and many more drug candidates targeting various complement components are currently undergoing evaluation in clinical trials (2). As a result, complement diagnostics has become increasingly important in the clinic. Accurate and comprehensive analysis of complement activity is crucial to diagnose, manage, and treat complement-related disorders. However, accurate determination of complement status has proven to be challenging, particularly within the constraints of routine clinical practice. Some of the challenges currently encountered include:

- Pathway complexity: The complement system consists of multiple components and three activation pathways that also interconnect with other immune pathways. Most diagnostic methods focus on a limited set of complement components, only providing a partial view of the cascade (Hurler et al.).
- Heterogeneity of complement-mediated diseases: The diversity of complement-related disorders makes it difficult to accurately identify and diagnose specific complement deficiencies, excesses, or abnormalities (3, 4).
- Patient heterogeneity: Patients with complement disorders exhibit substantial clinical diversity and variations in complement profiles. In addition, complement activation is highly dynamic and can change rapidly in response to stimuli (4). A snapshot measurement may not capture the full extent of complement activation. These variations not only complicate diagnostic accuracy but also affect treatment decisions, as a one-size-fits-all approach is often inadequate.
- Lack of sensitivity: Complement assays may lack the sensitivity to detect subtle changes in complement activity, especially in cases with partial complement deficiency or subtle dysregulation (4).
- Sample sensitivity: Many of the complement proteins are heat-labile and sensitive to post-sampling *in vitro* activation, which necessitates strict requirements for pre-analytical sample handling (5, 6).
- Lack of standardization: The absence of standardized methods, assays and reference materials, and uniform protocols for pre-analytical sample handling (collection, processing, and storage) can lead to inconsistent results obtained from different laboratories, hindering reliable comparisons (5–7).

This Research Topic provides a platform to critically discuss recent advancements in complement diagnostics and strategies for overcoming its challenges, offering recommendations for future research. This topic includes studies dedicated to unraveling the mechanisms underlying complement dysregulation and explores novel biomarkers relevant to complement-mediated diseases.

With regard to new biomarkers, Burgelman et al. summarize in their review article the current knowledge and explore future opportunities regarding complement proteins as biomarkers for neurodegenerative disorders such as Alzheimer’s disease (AD) and multiple sclerosis (MS). They suggest that assessing complement protein levels in biofluids could potentially serve as biomarkers for disease progression, severity, or responses to treatment in these diseases. The authors indicate that measuring ‘free’ complement components in blood/biofluids has several drawbacks, e.g. that the cellular origin of these free components is lost. In that light, they suggest measuring extracellular vesicles (EVs) offers an additional platform to enhance the diagnostic utility of complement markers. EVs can contain complement proteins, while the cellular origin can be identified through the presence of EV surface markers. This opens new directions for future research, as EVs are not (yet) thoroughly investigated as biomarkers for complement-mediated diseases.

Other studies on this topic investigated complement components as potential biomarkers for diseases such as COVID-19 (Bruni et al., Tierney et al.), rheumatoid arthritis (RA) (Matola et al.), kidney diseases (Zhang et al., Stea et al., Gastoldi et al.), type 2 diabetes and nonalcoholic fatty liver disease (NAFLD) (Habenicht et al., Dorflinger et al.), and cancer (Wang et al.).

The topic also includes studies presenting new methods or assays to improve the sensitivity and quality of the measurements (Meuleman et al., Hurler et al., Ye et al., Meinshausen et al., Stevens et al., Gastoldi et al.) and discusses the lack of standardization (Brandwijk et al., Michels et al.). Regarding new methods, Meuleman et al. and coworkers argue in their review for assays capable of analyzing complement deposits on cultured endothelial cells incubated with pathologic human serum. They propose this *ex vivo* test as a promising method for assessing complement activation, as it resembles the physiological context. This assay has been used to study complement activation in various diseases, and in some cases, to adjust treatment with complement inhibitory drugs. However, the authors also mention that there is no international standard for these assays and that the mechanisms for activation are not fully understood. Moreover, primary cell culture is challenging and hampers the development of a standardized commercial assay. The lack of standardized assays and reference materials not only holds true for assays using primary cells but is a challenge for complement diagnostics in general. This concern is also discussed by (Michels et al.). Their review focuses on C3 nephritic factor (NeF), a well-established biomarker for rare complement-mediated kidney disorders, but measuring NeFs for diagnostic purposes remains difficult. The authors discuss the diseases linked to NeFs, the diverse mechanisms of action seen in various NeF types, a number of laboratory techniques, and initiatives to establish standardized approaches.

Advances in complement diagnostics have the potential to improve the diagnosis and treatment of complement-mediated diseases, and it is a battle on two fronts. First, a continuous strive for novel achievements in the scientific forefront using advanced methodology and unraveling novel biomarkers. Second, and equally important, is to make complementary diagnostics accessible for everyday use in clinical and research settings. Addressing these challenges will require increased collaboration between researchers, clinicians, and industry partners.

## Author contributions

PN: Conceptualization, Investigation, Writing – review & editing. LS: Conceptualization, Investigation, Writing – review & editing. ET: Conceptualization, Investigation, Supervision, Writing – original draft.
